# Treatment of Intracranial Aneurysms Using the New Silk Vista Flow Diverter: Safety Outcomes at Short-Term Follow-Up

**DOI:** 10.3389/fneur.2021.713389

**Published:** 2021-07-09

**Authors:** José M. Pumar, Antonio Mosqueira, Jorge Olier, Claudio Rodriguez-Fernandez, Pedro Vega, Eva Gonzalez-Diaz

**Affiliations:** ^1^Neuroradiology Department, Catedra de Neurorradiología Intervencionista, Universidad de Santiago de Compostela, Santiago, Spain; ^2^Neuroradiology Department, Hospital Clinico Universitario de Navarra, Pamplona, Spain; ^3^Neuroradiology Department, Hospital Universitario Fundación Jimenez Diaz, Madrid, Spain; ^4^Neuroradiology Department, Hospital Universitario Central de Asturias, Oviedo, Spain; ^5^Neuroradiology Department, Hospital Universitario de Cruces, Barakaldo, Spain

**Keywords:** flow-diverter, aneurysm, embolization, silk vista, stent

## Abstract

**Background:** Flow diverters are widely used as the first endovascular treatment option for complex brain aneurysms due to their high percentage of occlusion and low morbi-mortality. The Silk Vista device is a new generation of flow diverters designed to facilitate full visibility, improve apposition to the vessel wall, and enhance navigability. Indeed, its greatest advantage is that it enables the easier navigation of stents between 3.5 and 4.75 mm through a 0.021 microcatheter. The objective of this study was to evaluate the safety and effectiveness of Silk Vista systems for treating cerebral aneurysms.

**Methods:** This prospective observational study included 25 consecutive patients with 27 wide-necked unruptured aneurysms treated with SILK Vista who were retrospectively analyzed for safety and efficacy.

**Results:** Endovascular treatment was successfully performed in all patients. The final morbidity and mortality rates were both 0.0%. Short-term (3–5 months) angiographic follow-up revealed 21 complete occlusions and 6 near-complete occlusions. No significant parent artery stenosis was observed.

**Conclusions:** This report demonstrates the efficacy of Silk Vista in treating brain aneurysms, although longer experiences should be carried out to confirm our results.

## Introduction

The introduction of flow diverter stents represented a new treatment option for those cerebral brain aneurysms that could not be managed with the usual endovascular techniques (1–3).

Since the Food and Drug Administration (FDA), in 2011, authorized the use of the first Flow diverter (Pipeline Embolization Device, Medtronic, Dublin, Ireland), flow diverters (FDs) began to be used representing an important option in the treatment of large and long brain aneurysms, obtaining satisfactory results both in the degree of occlusion and in the clinical efficacy (1–8). The neurovascular community has been progressively increasing their use, developing a new-generation the FDs, with different structures, in order to produce better parent artery reconstruction and improve endothelial cells formation across the aneurysm neck (Silk flow diverter, Balt Extrusion, Montmorency, France; FRED, MicroVention, Tustin, CA; Derivo Embolization Device, Acandis GmbH, Germany; Surpass stent, Stryker Neurovascular, Kalamazoo, MI, USA; p64, Phenox GmbH, Bochum, Germany) (9–14).

Recently, novel devices have been introduced several systems have been designed in order to reduce thromboembolic complications, facilitate navigability, improve radial force and radiopacity, including the development of FDs specifically designed for the treatment of aneurysms beyond the Willis Circle (PED Shield, Medtronic, Dublin, Ireland; p48, Phenox GmbH, Bochum, Germany; Silk Vista Baby, Balt Extrusion, Montmorency, France; Fred Junior, MicroVention, Tustin, CA, USA) (15–21).

The SILK Vista (SV) system represents a new generation of Silk flow diverters with a redesigned delivery system. Its greatest advantage is that it allows a better navigation and more controlled stent delivery through a 0.021 microcatheter, presenting a full radiopacity and resheathability capacity after deployment of up to 90%. We report our preliminary results using this device in the management of brain aneurysms.

## Materials and Methods

### The SILK Vista System

**Figure 1:**
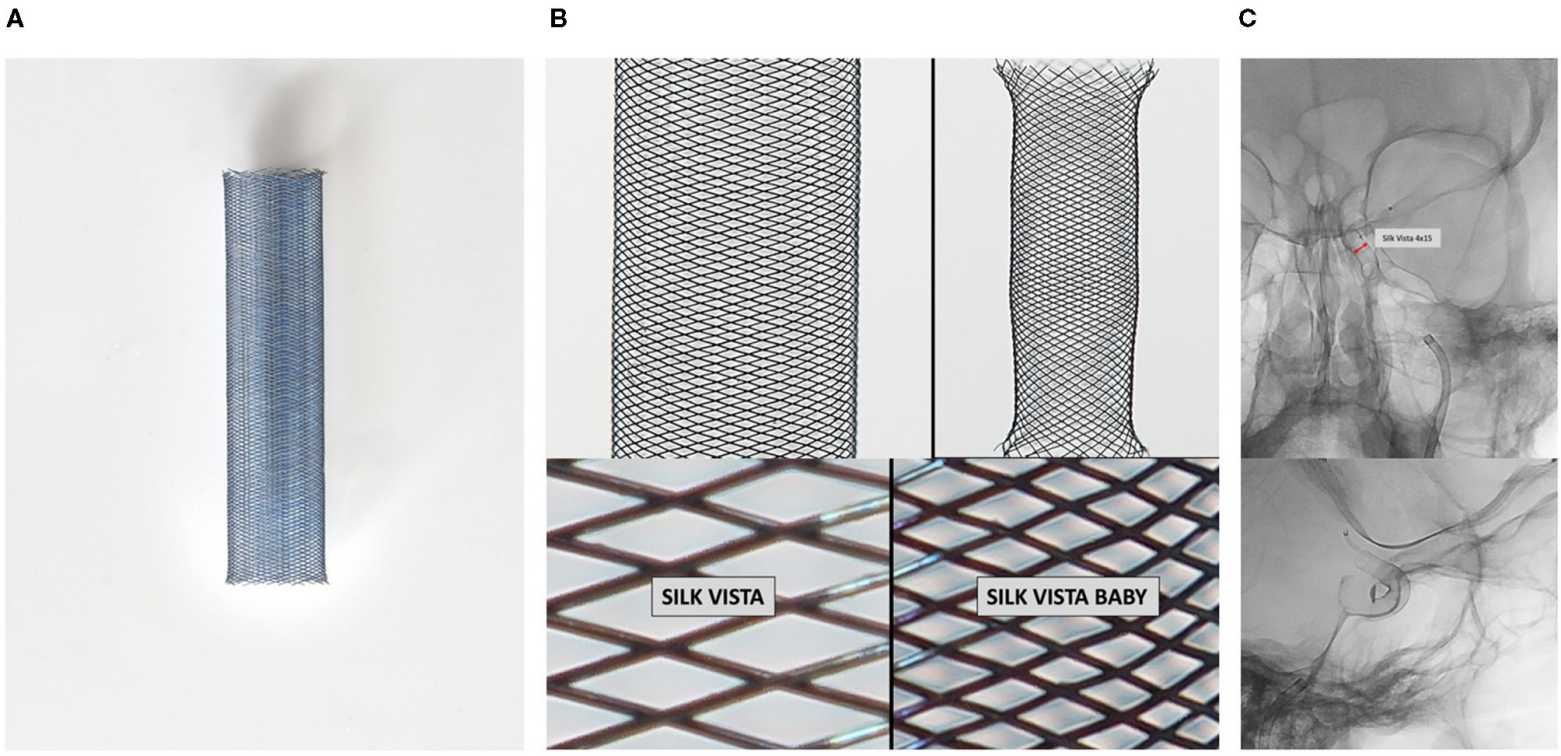
Schematic graphic representation of the SV delivery system. **(A)** SV appearance fully open; **(B)** comparison between SV and SVB, both with 48 DFT. The SV, however, are three times less dense and no present flared ends. **(C)** Good radiopacity SV. SV, Silk Vista; SVB, Solk Vista Baby.

The SV is a novel self-expanding stent designed to treat complex brain aneurysms that received the CE mark on June 6, 2020. The SV contains 48 drawn filled tubing (DFT) Nitinol/platinum wires, of a slightly larger caliber and a mesh density three times less dense than the Silk Vista Baby (SVB). This technology facilitates a good radiopacity and the precise controlled deployment of the entire device (Figures 1A,B).

This device and the p64 MW HPC are the only systems designed to treat vessels between 3 and 5 mm in diameter that navigate through a microcatheter of 0.021 in diameter. SV is currently available in 26 references, with a length between 15 and 40 mm and a diameter between 3.5 and 4.75 mm.

The stent is folded in a plastic sheath, the distal end of which is protected by a metal cannula that allows it to be safely inserted into the microcatheter. The delivery system presents an improved pusher profile to achieve the best compromise between flexibility and pushability. It has a delivery wire of nitinol with a 9-mm distal atraumatic platinum coil at a 45° angle.

Its design is similar to that of a SVB with the same instructions, precautions, and technical features (Figure 1), except that it has no present flared ends, which, together with its greater radial force, facilitate its better apposition to the vessel wall.

### Patient Population

This was a prospective observational study that included 25 consecutive patients with 27 wide-necked aneurysms treated with SV system at four Spanish university tertiary care centers, each of whom had experience with more than 30 SILK plus implanted. This study was approved by the institutional ethics committee. Written informed consent was obtained from all patients.

Patients between 18 and 80 years of age, with a pre-treatment modified Rankin scale (mRS) of 0–2, anterior circulation wide-neck unruptured aneurysm, or beyond 30 days since the hemorrhage, regardless of prior treatments, were included. Exclusion criteria included the presence of hemodynamically significant atherosclerotic lesions in the carotid artery on the same side, intolerance to heparin or resistance to antiplatelet therapy, coagulopathies, and an abnormal platelet count. The indication for endovascular treatment was made by a vascular neuroscience team of neurologists, neuroradiologists, and neurosurgeons.

### Data Collection and Follow-Up

We recorded each patient's demographic data, clinical presentation, aneurysm location, size of the SV, clinical and radiological follow-up information, and adverse events Clinical status was assessed during hospitalization, at discharge, and at short-term (3–5 months), using the modified Rankin scale (mRS). Any decrease in grade on the mRS scale was identified as morbidity.

Angiographic follow-up was performed at short-term (3–5 months). The imaging was reviewed and compared by two senior endovascular neurosurgeons who were not involved in the procedure for initial and follow-up occlusion grades. The degree of aneurysm occlusion was assessed according to the O'Kelly Marotta (OKM) grading scale (22).We considered grade C or D as a satisfactory outcome.

### Endovascular Procedure

All procedures were performed under anesthesia. All patients received double antiplatelet therapy before and after treatment as well as complementary heparinization according to our protocol for brain stenting (9).

**Figure 2:**
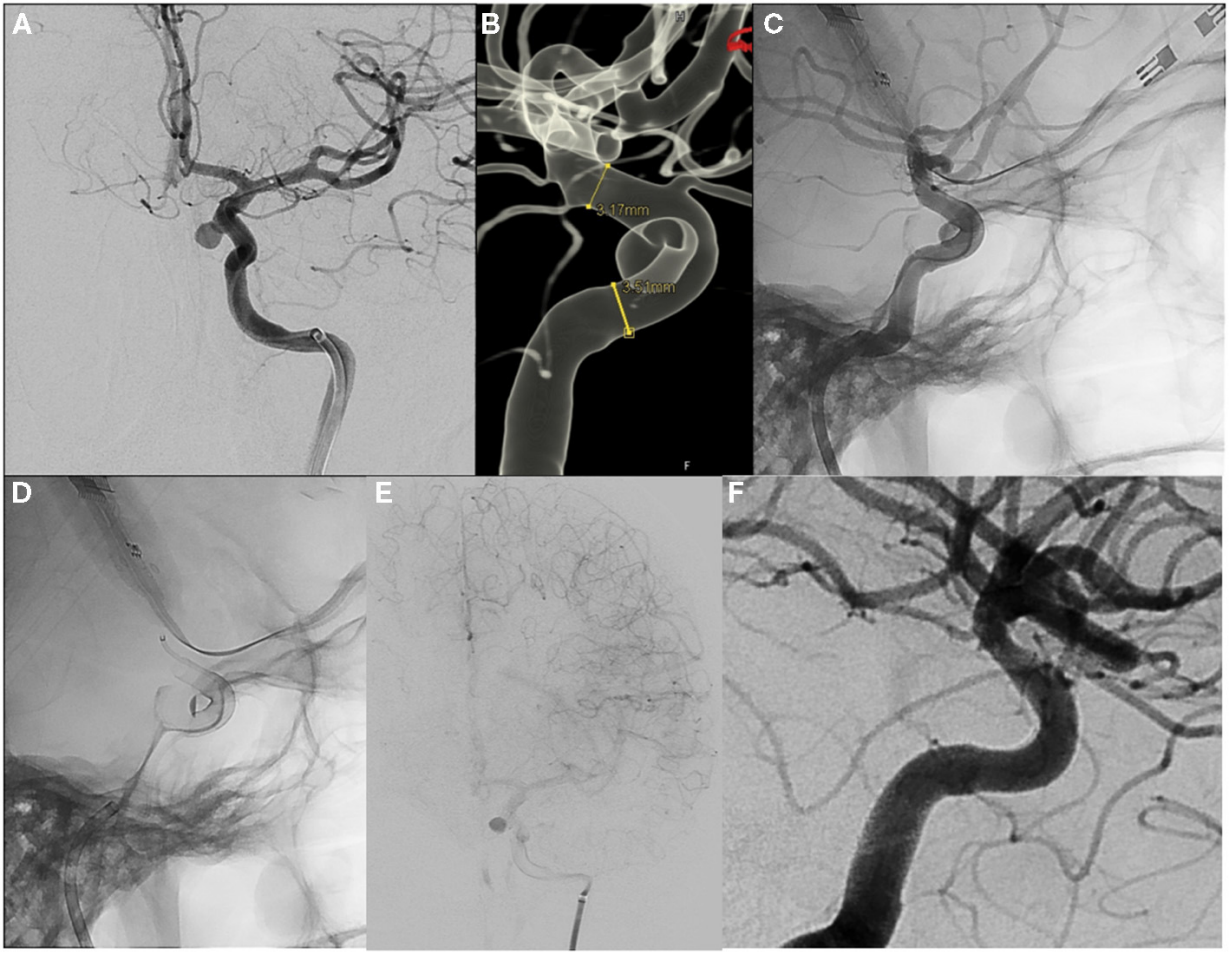
DSA and reconstruction of 3D rotational of the right internal carotid artery **(A,B)** show a paraclinoid aneurysm. Silk Vista device 3.75 × 20 mm is deployed through the Rebard 18 microcatheter, covering the neck of the aneurysm with a safety margin **(C)**, after which the microcatheter is advanced through the interior of the stent **(D)**. Postoperative angiography reveals a flow reduction **(E)**. The 4-month follow-up DSA shows complete occlusion of the aneurysm **(F)**.

The SV deployment strategy aimed at strict compliance with the recommendations of the SV manufacturer (Balt Extrusion, Montmorency, France). The choice of SV was selected by three-dimensional angiography, and the stent length was determined to be 30% longer than the aneurysm neck overlapping both sides of the aneurysm neck, by at least 4 mm. The diameter was chosen by oversizing the proximal diameter of the parent artery by 0.25 mm. Selection of the FDs, deployment of multiple devices, and implementation of adjunctive coiling were carried out at the discretion of the individual neurointerventionalist (Figures 2A–F). In cases in which additional aneurysm coiling was performed, a microcatheter was initially positioned inside the aneurysmal sac, followed by semi-jailing of the microcatheter between the parent vessel wall and the FDs on deployment.

Deployment failure was assessed in terms of the following aspects: failure to advance through the delivery catheter, poor opening, poor positioning, shortening, and stent displacement.

### Statistical Analysis

Data are presented as means and SD for continuous variables and as numbers and percentages for categorical variables. Statistical analyses were performed using a Student's *t*-test, Chi-square test, Mann-Whitney test, ANOVA, and multivariate analysis using IBM^®^ SPSS^®^ statistics v. 22 (IBM Co., Armonk, NY, USA). For statistical analyses, values of p < 0.05 were considered statistically significant.

## Results

### Patient and Aneurysm Characteristics

Between August 15, 2020, and November 31, 2020, 25 patients (11 female, 14 male; mean age, 58-years-old; age range, 32–80) with 27 aneurysms who fulfilled the criteria of inclusion were enrolled in our study. Angiography follow-ups were carried out prior to February 2020; data locking took place in March 2020.

Aneurysms locations were: cavernous internal carotid artery (ICA; n.4), paraophthalmic ICA (n.8), superior hypophyseal artery (n.5), paraclinoid ICA (n.2), supraclinoid ICA n.5), posterior communicating artery (PcomA; n.2), and anterior cerebral artery (ACA; n.1).

Four aneurysms had previously ruptured and had recurrence after initial coiling. Two patients had two aneurysms. The aneurysm size ranged from 2 to 16 mm (median, 7 mm). The neck size ranged from 1.5 to 10 mm (average, 4 mm). The neck to sac ratio ranged from 0.3 to 1 (average 0.8).

### Intraprocedural Technique

The Silk Vista advanced easily into a Rebard 18 microcatheter (Medtronic) in all cases. Stent deployment was successfully achieved in all patients. One patient was implanted with two devices. The mean procedure duration was 92.7 min (range, 40–235 min), and the mean cumulative fluoroscopy time was 31.4 min.

We implanted 26 SV in 25 patients. Each patient was treated using a single SV, except in one patient with a large paraophthalmic aneurysm, where the length of the SV was underestimated, who was treated with two overlapping stents. In three cases, a semi-jailing technique was used for coiling. In two patients, with tortuous vascular anatomy, the adaptation of the SV to the vessel in its middle part was not complete, and an angioplasty (PTA) was performed with a Scepter balloon (MicroVention, Tustin, CA) to fully open the stent and improve its apposition to the vessel. A postoperative VasoCT showed excellent wall apposition in both cases.

### Immediate Angiographic and Clinical Results

Following FD deployment, 22 aneurysms showed contrast stasis in the venous phase. No aneurysm showed the complete absence of contrast fill (OKM grade D), 13/27 (%) showed persistent filling (OKM grade B), and 14/27(50%) showed complete filling (OKM grade A).

No periprocedural thrombus formation was observed. No new neurological deficits developed after endovascular treatment in any of the patients, and no bleeding or ischemic events occurred during or after the endovascular treatment.

### Clinical and Imaging Follow-Up

Clinical and imaging follow-up data were available for all 25 patients at a mean of 3.45 ± 0.8 post-procedure months (range, 3–5). No new neurological deficits were observed in any patient.

Short-term anatomical results revealed a complete aneurysm occlusion (OKM D) in 21/27 (77.7%) aneurysms. A near-complete occlusion (OKM C) was detected in another 6/27 (22.3%) aneurysms. No cases of in-stent stenosis or in-stent thrombosis were observed.

**Figure 3:**
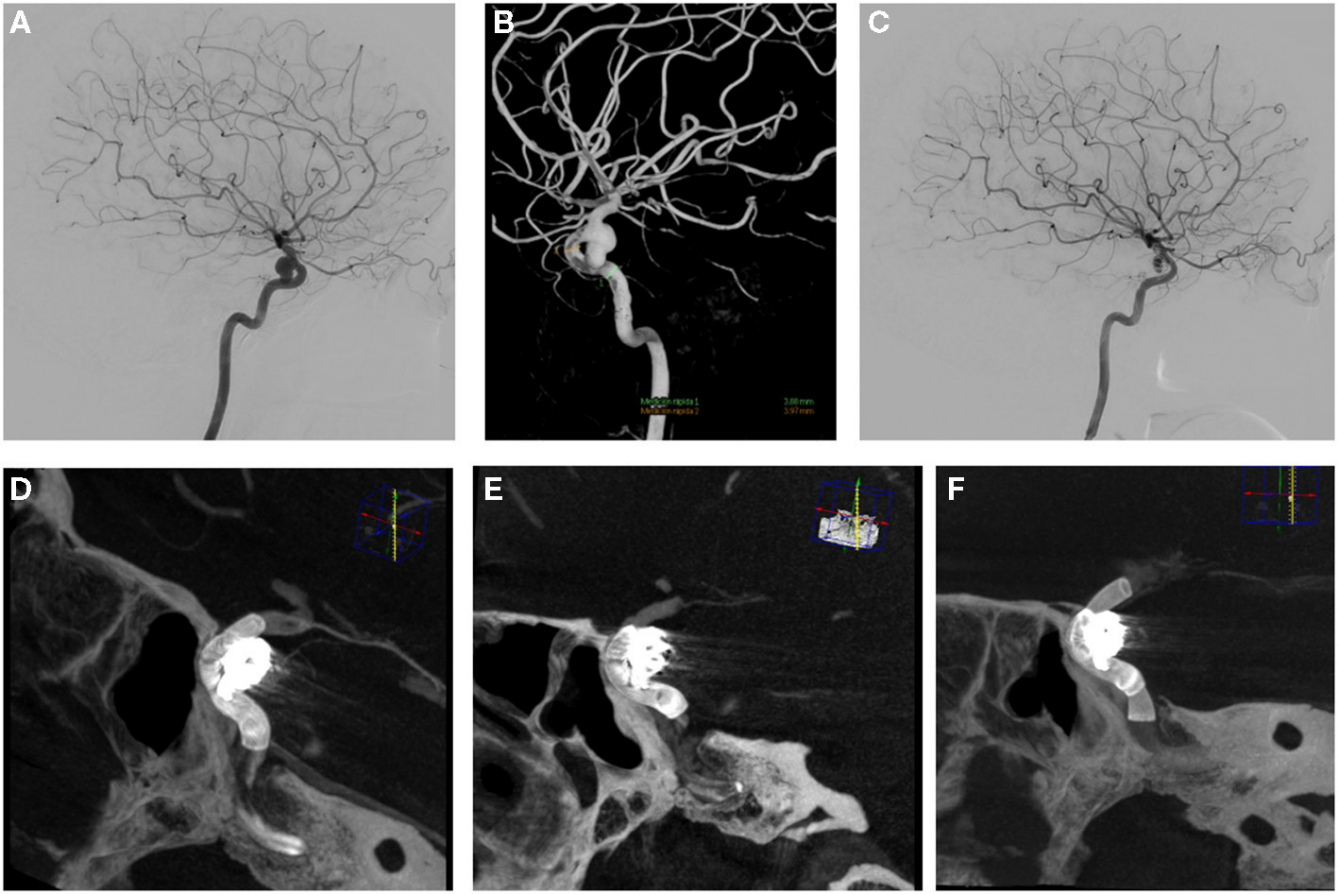
A 52-year-old woman referred for embolization of an incidental aneurysm. Digital subtraction angiography **(A)** and 3D rotational angiography **(B)** show a wide-neck left para-ophthalmic aneurysm. Stent deployment (SV 4 × 20 mm) and compartmental coiling (semi-jailing technique) was performed **(C)**. A Vaso CT shows adequate stent apposition to the vessel covering of the aneurysm neck **(D)**. A follow-up at 3 months, shows shortening of SV without completely covering the aneurysmatic neck **(E)**. An overlapping 4 × 25 mm SV was deployment covering of the aneurysm neck **(F)**.

In one case, delayed migration of the SV was detected. A 52-year-old woman presented an aneurysm in the left superior hypophyseal segment of the ICA (6.43 × 6.97 mm with a neck of 3.6 mm). The diameter of the petrous segment of the internal carotid artery was 4.1 mm, and the diameter of the paraclinoid segment was 2.7 mm. The aneurysm was treated with flow diversion and coiling. A 4 × 20-mm SILK Vista was deployed across the aneurysms without difficulty. Immediate post-procedure angiography control showed adequate SV placement with contrast stasis in the aneurysm (Figures 3A–D) and a VasoCT demonstrated adequate stent apposition to the vessel wall with covering of the aneurysm neck. The procedure and postoperative course were uneventful, and the patient was discharged the following day.

Three months later, angiography showed proximal displacement of the stent with partial filling of aneurysms. A VasoCT confirmed the complete non-coverage of the neck. The deployment of an additional stent was necessary, and an overlapping 4 × 25-mm SV was subsequently deployed beyond the first stent to bridge the neck of the aneurysm and disrupt the inflow jet (Figures 3E,F). A VasoCT subsequently performed demonstrated adequate apposition to the interior of the prior SV and to the vessel wall with covering of the aneurysm neck.

## Discussion

Some aneurysms have morphological characteristics that hinder conventional endovascular treatment, requiring the use of new systems such as in the form of FDs. Many studies have demonstrated the feasibility and effectiveness of FDs in the treatment of nearly all types of aneurysms, which are now widely accepted as a suitable alternative to conventional intracranial aneurysm treatment, especially for complex aneurysms (3, 6, 9, 12, 15, 19). This preliminary clinical experience of aneurysm treatment (26 SV implanted in 25 patients) with SV devices was technically successful in all cases and showed excellent feasibility, safety, and short-term efficacy at the 3–5 month follow-up.

Although the short- and mid-term results after FD use have demonstrated good aneurysm occlusion as well as low morbidity and mortality, one of the major limitations of the FD implantation technology between 3 and 5 mm in diameter is that most systems are compatible with microcatheters with a lumen > 0.027 inches (Silk + -Balt-extrusion; Pipeline Vantage-Medtronic; FRED Jr. -Microvention; DERIVO mini –AcandisGmbH; P48 MW -Phenox).

In 2020, a redesigned “third-generation SILK,” called the SILK Vista was introduced, representing the most significant technical improvement, being a FD for vessels diameters between 3 and 5 mm compatible with 0.021 inner diameter microcatheters, which facilitates the navigability of the system while minimizing technical complications during treatment.

Previous experiences using first-generation silk devices had relatively high morbidity and mortality rates (2, 3, 9, 23–25), resulting in specific problems mainly related to deployment difficulties. In these studies, the rate of successful deployment reported varied between 75 and 96%, with an average of 88.6%, which contrasts with the rate of technical feasibility of 100% reported in our results and those reported in other series with P48-MW (20, 21). However, a direct comparison between our results and the reported results is not possible given the variations in key factors, such as patient selection criteria and population size. We believe that such differences are due to the fact that significant experience in the management of the silk system is needed to implant SV devices, as such a procedure is slightly different than that followed for the implantation of other FDs stents.

To date, no published short-term aneurysm occlusion or clinical outcome data exist with regard to the SV systems. Recently, Martínez-Galdámez et al. (26) described the first experience assessing, in a retrospective revision, the technical success and procedural safety in the management of a consecutive succession of 57 patients with 60 brain aneurysms between September 2020 and January 2021. In a prospective study, we report our experience assessing both the procedural safety of the system and its effectiveness in a clinical and radiological follow-up at short-term (3–5 months) in a case series of patients treated with SV system. This clinical study was carried out to conduct a *post-hoc* multicenter study.

In this preliminary clinical experience, aneurysm treatment (26 SV implanted in 25 patients) with SV deployment was technically successful in all cases. It is great percentage of success in deployment is probably related to the FD construction design, which enables the visibility of the entire device, better apposition to the vessel wall, and compatibility with a 0.021 delivery microcatheter, facilitating navigability. Our preliminary impression compared with the other current FDs is that it is technically simpler to deliver, deploy, maneuver, and perform crossings, presenting much lower friction, more flexibility, and better radiopacity.

In two patients with very tortuous anatomy, immediate post-deployment control angiograms showed an incomplete opening of the SV in its middle part without a good wall apposition, and an in-stent PTA was performed with excellent wall apposition in both cases. The 8% post-deployment angioplasty rate reported was similar to 5.6% of PED and markedly lower than 28% for Surpass and although malapposition is still a complication of the system, new FDs (FRED; P48 MW HPC; P64 MW HPC; SV.)have been specifically designed provide higher radial forces and better navigation systems to facilitate wall apposition in difficult opening situations.

According to our experience, adequate stent opening is achieved only when the stent is slowly deployed from the microcatheter, and it is also important to ensure that the stent is steadily placed at the vessel wall by pushing the microcatheter distally through the stent; we have generally avoided excessive compaction or stretching of the SV during deployment to reduce the risk of damaging the flow diverter.

In this experience, we have reported one case of delayed shortening or migration of the SV in the angiography follow-up performed at 3 months, evidencing that the neck of the aneurysm is no longer covered by the device. Lubicz et al. (23) previously described the delayed migration of a Silk stent in a patient with a giant saccular carotid ophthalmic aneurysm. Although Chalouhi et al. (27) reported 5 cases of FD migration or shortening with serious and potentially fatal complications in our case, the patient did not present any clinical complications. There are various potential mechanisms to be contemplated which might cause FD shortening or migration, involving the undersizing the caliber of the stent in relation to the diameter of the target vessel, substantial diameter differences of the target vessel diameters, faulty wall apposition of the FD, and excess manipulation of the device during implantation. In our case, we believe that due to the vascular tortuosity, the radial force of the stent exerted a non-uniform pressure on the wall of the vessel, facilitating the displacement toward the proximal part of greater caliber.

At the 3-month angiographic follow-up, 21 aneurysms (77.7%) showed complete occlusion (OKM score D), and 6 aneurysms showed near-complete occlusion (OKM score C). This rate is comparable with the rates reported in the literature (10, 13, 17), such as those reported by Pierot et al. (28) in the SAFE study, where an adequate occlusion rate of 81.1% was reported using the FRED device. Although the sample size was small, and the follow-up period was short, SV system appear to be a safe and effective form of treatment for unruptured ICA aneurysms. Based on our results, we believe that a prospective multicenter study to validate the safety and effectiveness of the SV stents would be worthwhile.

The major limitations of this prospective study include the limited cohort of 25 patients, a short-term follow-up period of 3-5 months. Another major limitation is the heterogeneity of the aneurysms and the fact that the majority of aneurysms were located in the ICA, which supposes a lower risk of complications compared with those located elsewhere in the anterior circulation.

## Conclusion

Based on the results obtained in this study with a small series of cases the efficacy of Silk Vista seems to be promising as a flow diverter with special characteristics, enabling easy deployment with low friction, very good radiopacity, predictable opening, and operator-friendliness. To confirm the apparent high safety of the device, long-term follow-up and a larger cohort is necessary to determine this.

## Data Availability Statement

The original contributions presented in the study are included in the article/supplementary material, further inquiries can be directed to the corresponding author.

## Ethics Statement

The studies involving human participants were reviewed and approved by Comite etico del SERGAS. The patients/participants provided their written informed consent to participate in this study.

## Author Contributions

JP, AM, and JO: conception and design of the study. JP, CR-F, EG-D, and AM: data acquisition and analysis. CR-F and JO: manuscript drafting. EG-D and JO: critical revision for important intellectual content. All authors have reviewed and approved the manuscript.

## Conflict of Interest

JP was a consultant for Balt. The remaining authors declare that the research was conducted in the absence of any commercial or financial relationships that could be construed as a potential conflict of interest.
